# Neuropeptide FF indirectly affects testicular morphogenesis and functions in medaka

**DOI:** 10.1073/pnas.2209353119

**Published:** 2022-11-07

**Authors:** Soma Tomihara, Kana Ikegami, Rinko Shimomai, Chie Umatani

**Affiliations:** ^a^Department of Biological Sciences, Graduate School of Science, The University of Tokyo, Bunkyo-ku, Tokyo 113-0033, Japan;; ^b^Nagahama Institute of Bio-Science and Technology, Nagahama, Shiga 526-0829, Japan;; ^c^Department of Aquatic Bioscience, Graduate School of Agricultural and Life Sciences, The University of Tokyo, Bunkyo-ku, Tokyo, Japan, 113-8657;; ^d^Division of Applied Biological Chemistry, Graduate School of Agriculture, Tokyo University of Agriculture and Technology, Fuchu-shi, Tokyo, 183-8509, Japan

**Keywords:** testis, neuropeptide FF, teleost

## Abstract

Testicular morphogenesis and functions are considered to be under the control of neural and endocrine systems. However, the available literature is mainly limited to mammals, and it remains unclear how they are regulated in teleost species. Here, we demonstrated that neuropeptide FF (NPFF) in the brain is responsible for the follicle-stimulating hormone expression in the pituitary, which facilitates the testicular morphogenesis and androgen synthesis, and subsequently contributes to successful spermatogenesis. The present findings give us important insights into the neuroendocrine regulatory mechanisms underlying the testicular morphogenesis and functions in teleosts.

Testicular morphogenesis and functions are generally considered to be regulated by the hypothalamic-pituitary-gonadal axis in vertebrates (1–3). In mammals, it is widely accepted that gonadotropin-releasing hormone (GnRH) from GnRH neurons in the hypothalamus induces the release of follicle-stimulating hormone (FSH) from the pituitary, which facilitates spermatogenesis in testis (4). However, in teleosts, hypophysiotropic GnRH-knockout (KO) males show normal testes in medaka (5). It has also been reported that FSH- or FSH receptor–KO males also show normal testes in teleosts (5–7). Thus, the regulatory mechanism of testicular functions in teleosts has not been well understood. Here, we focused on an RFamide peptide as a candidate regulator of testicular functions, since it has been suggested that besides GnRH, some RFamide neuropeptides (neuropeptides with C-terminal Arg-Phe-NH_2_ motif) are involved in spermatogenesis or Sertoli cell maturation in mammals (8, 9). Furthermore, this neuropeptide family is conserved among vertebrate species (10). We hypothesized that RFamide neuropeptides play a key role in testicular functions in teleosts and analyzed the functions of an RFamide called neuropeptide FF (NPFF) in a teleost medaka by using *npff*-KO males.

## Results

**Fig. 1.**
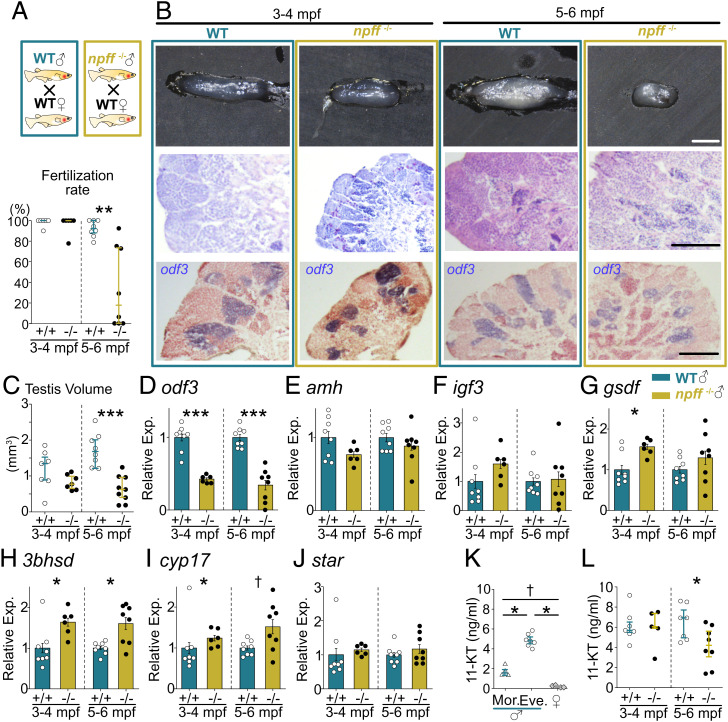
The testes *of npff^−/−^* are abnormal, resulting in the decreased fertilization rate. (*A*) Fertilization rate of WT and *npff^−/−^*. (*B*) Testicular morphology (*Upper Row*: whole testis; *Center Row*: HE-stained histology) and ISH of *odf3* (*outer dense fiber of sperm tails 3*: purple signals) in WT and *npff^−/−^* (*Bottom Row*). (Scale bars, 1 mm [*Upper Row*], 100 µm [*Center and Bottom Rows*].) (*C*) Testicular volume of WT and *npff^−/−^*. (*D–J*) Gene expression levels in WT and *npff^−/−^*. (*D*) *odf3*, (*E–G*) Sertoli cell–specific genes, (*E*) *amh* (anti-Müllerian hormone), (*F*) *igf3* (insulin-like growth factor-3), (*G*) *gsdf* (gonadal soma–derived factor), (*H–J*) Leydig cell–specific genes, (*H*) *3bhsd* (3β-hydroxysteroid dehydrogenase), (*I*) *cyp17* (17α-hydroxylase), and (*J*) *star* (steroidogenic acute regulatory protein). (*B–K*) All testes were sampled in the evening. (*K*) Blood concentration of 11-KT in WT males (morning, evening) and females (evening). (*L*) 11-KT concentration in WT and *npff^−/−^* males in the evening. ^†^*P* < 0.1: *P < 0.05, **P < 0.01, ***P < 0.001.

We found that *npff*-KO (*npff*^−/−^) males showed a low fertilization rate (Fig. 1*A*) and small testes at 5 to 6 months post fertilization (mpf) (Fig. 1 *B*, *Upper Row*, and *C*). We observed the testicular architecture in hematoxylin and eosin (HE)–stained preparations. We found that *npff*^−/−^ testes showed normal testicular structure (Fig. 1*B*, *Center Row*), although they were smaller than those of wild type (WT), particularly at 5 to 6 mpf (Fig. 1 *B*, *Upper Row*, and *C*). In addition, *in situ* hybridization (ISH) revealed that *odf3*, a mature sperm marker (11), was expressed even in the *npff*^−/−^ testes at all ages (Fig. 1*B*, *Bottom Row*), indicating that *npff*^−/−^ testes can produce mature sperms regardless of their small size. We then performed quantitative PCR (qPCR) analysis and found that the expression level of *odf3* in *npff*^−/−^ testes was lower than that in WT (Fig. 1*D*). To further examine the effects of *npff* loss on the testicular functions, we analyzed the expression of Sertoli/Leydig cell–specific genes (Fig. 1 *E*–*J*). *npff*^−/−^ showed a significant increase in *3bhsd* and *cyp17* expressions (Fig. 1 *H* and *I*), which are genes for androgen synthetic enzymes. We also examined the blood concentration of 11-ketotestosterone (11-KT), which is an essential sex steroid hormone for spermatogenesis in fish (12). Since WT male medaka showed time-of-day changes in 11-KT concentrations, higher in the evening than in the morning (Fig. 1*K*), we collected blood in the evening and compared 11-KT concentrations between WT and *npff*^−/−^. The 11-KT concentration of 5 to 6 mpf *npff*^−/−^ blood was lower than that of WT (Fig. 1*L*).

**Fig. 2.**
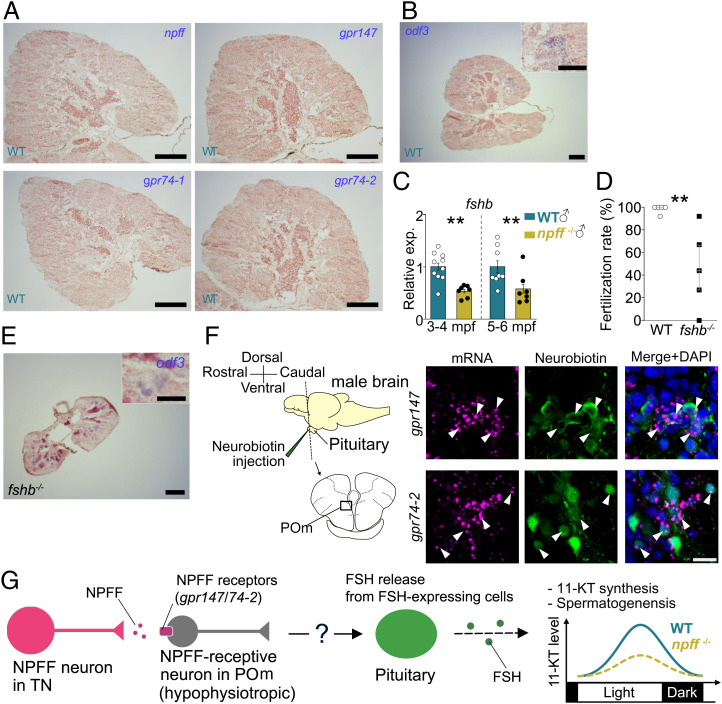
NPFF signal via hypophysiotropic neurons is responsible for the *fshb* expression in the pituitary, which can affect the testis morphology. (*A*) Signals of *npff* and its receptor genes were not observed in testis. (*B*) *odf3* signals were detected in the same experiment as (*A*). (*C*) *fshb* expression in the pituitaries of WT and *npff*^−/−^ males. (*D*) Fertilization rate of WT and *fshb*^−/−^ males at 5 to 6 mpf. (*E*) *fshb*^−/−^ testis at 5 to 6 mpf. (Scale bars, 100 µm and inset 50 µm.) (*F*) Images of neurons double labeled by probes for *gpr147*/*74-2* mRNA (magenta) and neurobiotin injected from the pituitary (green) in the POm of WT males. There were some neurons colabeled with *gpr147*/*74-2* mRNA signals and neurobiotin (arrowheads). (Scale bar, 10 µm.) Samplings were performed in the morning (*A*, *B*, and *D*–*F*) and in the evening (*C*). (*G*) Schematic illustration of NPFF-induced modulation to the testicular functions. NPFF facilitates FSH expression via hypophysiotropic NPFF receptor–expressing neurons, resulting in high levels of 11-KT in the evening and potentially contributing to the appropriate spermatogenesis. TN, terminal nerve. **P < 0.01.

Next, to examine whether NPFF directly affects testicular functions, we performed ISH of *npff* and genes for NPFF receptors (*gpr147*, *74-1*, and *74-2*). In the testis, signals for *npff* and all of the NPFF receptors were not observed (Fig. 2*A*), whereas those for *odf3*, which served as a positive control, were detected (Fig. 2*B*). Since FSH from the pituitary facilitates spermatogenesis in mammals, we further analyzed fsh beta-subunit gene (*fshb*) expression in WT and *npff*^−/−^ pituitaries. We detected significantly lower *fshb* expression in *npff*^−/−^ pituitaries (Fig. 2*C*). In addition, 5 to 6 mpf FSH-KO (*fshb*^−/−^) males showed lower fertilization rates (Fig. 2*D*) and smaller testis (Fig. 2*E*) compared with WT (Fig. 2*B*). Furthermore, retrograde labeling of hypophysiotropic neurons demonstrated that a part of *gpr147*/*74-2*-expressing neurons in the POm (the area preopticus pars magnocellularis) projected to the pituitary (Fig. 2*F*).

## Discussion

The present study demonstrates that NPFF plays a crucial role in testicular morphogenesis and functions by increasing the expression of *fshb* in the pituitary, because *npff*^−/−^ males showed a low fertilization rate (Fig. 1*A*), small testes (Fig. 1 *B* and *C*), and low *fshb* expression in the pituitary (Fig. 2*C*). We have previously shown that NPFF is specifically expressed in the terminal nerve in the teleost brain and the NPFF neurons project their axons broadly in the brain but not the pituitary (13, 14), and here we showed that NPFF receptors (*gpr147*/*74-2*) expressing neurons in the POm projected to the pituitary (Fig. 2*F*). Taken together, it is conceivable that NPFF modulates *fshb* expression via neurons expressing NPFF receptors in the POm (Fig. 2*G*). It should be noted that FSH- or FSH receptor–KO males have been reported to demonstrate normal testes (3–5). In the present study, experiments using older fish (at 5 to 6 mpf) than those in the previous studies (at ∼3 mpf) enabled us to find that FSH is involved in the testicular functions, at least in medaka.

Interestingly, 11-KT levels of WT males showed the difference between morning and evening (Fig. 1*K*), suggesting that there is a diurnal fluctuation of 11-KT levels in WT males. Also, our results showed that *npff*^−/−^ males showed lower 11-KT levels in the evening (Fig. 1*K*), lower expression of *odf3* (Fig. 1*D*), and a lower fertilization rate (Fig. 1*A*), which suggests that sufficient levels and/or diurnal fluctuation of testosterone play a role in appropriate spermatogenesis in teleost species. It should be noted that *npff*^−/−^ showed higher expression of *3bhsd*/*cyp17* (Fig. 1 *H* and *I*), which contradicts the results of lower 11-KT levels. It is conceivable that these steroidogenic genes are up-regulated to compensate for the deficiency of FSH signaling caused by *npff* KO, but it is insufficient to reinstate 11-KT level due to the small size of the KO's testis.

In summary, our present study suggests that NPFF affects testicular morphogenesis and functions, as illustrated in Fig. 2*G*. Since we demonstrated NPFF function in males, further analysis of NPFF in females will also help understanding teleost reproduction. In teleosts, the neuroendocrine mechanism regulating testicular function has been relatively unraveled compared to that of the ovary. Our study may advance the understanding of brain functions for successful reproduction in teleost males.

## Materials and Methods

We kept and used d-rR WT, *npff^−/−^* and *fshb^−/−^* medaka (*Oryzias latipes*) as described by Umatani et al. (14) and Takahashi et al. (5). All of the experiments were conducted in accordance with the protocols approved by the animal care and use committee of the Graduate School of Science, University of Tokyo (permission no. 20–6). Detailed procedures for other analyses are described in *SI Appendix*.

## Supplementary Material

Supplementary File

## Data Availability

All of the study data are included in the article and/or supporting information.
