# Supplementary material for: Neuropeptide FF indirectly affects testicular morphogenesis and functions in medaka
Source: Proc Natl Acad Sci U S A. 2022 Nov 7;119(46):e2209353119. doi: 10.1073/pnas.2209353119 (PMC9674221; doi:10.1073/pnas.2209353119)
Supplement: Supplementary File [file pnas.2209353119.sapp.pdf]

Materials and Methods

Animals

We used wild type (WT) d-rR medaka (*Oryzias latipes*), the gene-knockout line of *npff* ($npff^{-/-}$), which was generated by TALEN in Umatani *et al.*, 2022 (1), and the gene-knockout lines of *fshb* ($fshb^{-/-}$), which was generated by TALEN in Takahashi *et al.*, 2016 (2). We kept $npff^{-/-}$ line, while we obtained $fshb^{-/-}$ by crossing $fshb^{-/+}$ because $fshb^{-/-}$ female is infertile. They were kept at 27 ± 1°C with water circulation (Labreed, IWAKI Co., Ltd., Tokyo, Japan) under the following daylight condition: 14 hours light (8:00 AM-10:00 PM) and 10 hours dark. These fish were fed with artemia (Salt Creek Inc, Salt Lake, UT) or flake-food (Marubeni Nisshin Feed Co.,Ltd., Tokyo, Japan) for 2 to 5 times per day. The age of fish was described in the figure legends.

Sampling was performed as described below at two periods, the morning (9:00-12:00 AM) and the evening (3:00-6:00 PM).

Fertilization rate

The sexually mature WT or $npff^{-/-}$ males were maintained in pairs with sexually mature WT females for more than one week (until they showed consecutive spawning). In the morning, we collected eggs that attached on female abdomen and calculated fertilization rate by dividing the number of fertilized eggs by the number of total collected eggs.

Measurement of testis size

Since it is difficult to measure testis weight due to their smallness, we considered its shape as an approximate ellipsoid and estimated the volume as follows. In the evening, testes were excised from WT or $npff^{-/-}$ males, photographed using a digital camera (MC120HD; Leica Microsystems, Wetzlar, Germany), and calculated max rostrocaudal/dorsoventral lengths of testis from the images, which were regarded as major/minor axis of ellipsoid, respectively. The volumes were calculated according to the following formula.

$$\text{Estimated testis volume } (\text{mm}^3) = \frac{4}{3}\,(\text{rostrocaudal length (mm)})(\text{dorsoventral length (mm)})^2$$

Hematoxylin and eosin staining

For observing the testis morphology, we performed Hematoxylin and Eosin (HE) staining as described in previous study (2). In the evening, male medaka were deeply anesthetized with 0.1% eugenol (DS pharma Animal health, Osaka, Japan), and testes were removed and fixed overnight with Bouin's solution (picric acid saturated solution : formaldehyde : acetic acid = 15 : 5 : 1) at room temperature. After fixation, specimens were dehydrated with ethanol, permeated with xylene, and embedded in paraffin (Histprep568, FUJIFILM Wako Pure Chemical Co., Osaka, Japan). Then, they were sectioned serially at a thickness of 5 µm, deparaffinized, hydrated, and stained with HE. Sections were photographed by using a digital camera (DFC310FX; Leica Microsystems) attached to a DM 5000 B microscope (Leica Microsystems).

In situ hybridization

Sense and antisense digoxigenin (DIG)-labeled RNA probes for *odf3* (probe length: 852 bases) were generated through *in vitro* transcription from dsDNA obtained from testis cDNA.

For analyzing the gene expression of *npff* and their receptors, *gpr147*, *gpr74-1*, and *gpr74-2*, we used the probes that were also used in our previous study (1).

In situ hybridization were performed as described in the previous study (3) with minor modification for paraffin-embedded specimen. In the evening, male medaka were deeply anesthetized with 0.1% eugenol, and their testes were collected and fixed overnight with 4 % paraformaldehyde (PFA) in PBS (phosphate buffered saline). The specimen was prepared in the same methods up to hydration step for HE staining. Sections were postfixed with 4 % PFA in PBS for 10 minutes, treated with 2 mg/mL glycine in PBS for 5 minutes, and incubated with 0.25 % acetic anhydride in 0.1 M triethanolamine (Sigma-Aldrich, Darmstadt, Germany) for 10 minutes. Sections were subsequently hybridized overnight with DIG-labeled RNA probe at 58 °C. Hybridization signals were detected using alkaline phosphatase-conjugated anti-DIG antibody (Roche Diagnostics, Basel, Switzerland) and 5-nitro blue tetrazolium/bromo-4-chloro-3-indolyl phosphate (NBT/BCIP) (Roche) as chromogenic substrates. Photo images were acquired using the same equipment used in HE staining.

RT-qPCR

Reverse transcription and quantitative PCR (qPCR) protocol was performed as described in our previous study (1). In the evening, Male fish were deeply anesthetized with 0.1% eugenol, sacrificed, and extracted its pituitary and testis. Total RNA was purified using FastGene^{TM} RNA basic kit (Nippon Genetics co Ltd., Tokyo, Japan), eluted with 20 µL elution buffer, and subsequently reverse-transcribed by FastGene^{TM} Scriptase II cDNA Synthesis 5x Ready Mix OdT (Nippon Genetics). It should be noted that the concentration of total RNA was adjusted to 5 ng/µL in the analyses of testis, whereas max volume of total RNA in the pituitary was used for reverse transcription due to their low concentration. For real-time PCR, 1 µL of cDNA diluted with 10-fold MQ (testis) or 5-fold MQ (pituitary) was mixed with KAPA SYBR Fast qPCR kit and amplified with LightCycler 96 (Roche). The temperature profile of the reaction was 95 °C for 90 seconds, 45 cycles of denaturation at 95 °C for 10 seconds, annealing at 60 °C for 10 seconds, and extension at 72 °C for 10 seconds. The PCR product was verified using melting curve analysis (95 °C for 10 seconds, 65 °C for 60 seconds, and 97 °C for 1 second). The data were normalized to a housekeeping gene, elongation factor 1 (for testis) or ribosomal protein s13 (for pituitary). The primer sequences of each gene for amplification were the followings:

elof1	Forward	5’- CCATCACTTATCTGTCAGAGCCGG-3’
	Reverse	5’- ATGCTCATCCTCTCCACGTTCAC-3’
odf1	Forward	5’- GGTTCCTTTGGTCAGCATTT-3’
	Reverse	5’- AATAGGCCCTCTGGGTTTGT-3’
amh	Forward	5’- GAGACCTGGAGGTCACGTTC-3’
	Reverse	5’- GAGAGCTGCTGCTGGAAGAT-3’
igf3	Forward	5’- CGGAGACCGAGGCATCTAC-3’
	Reverse	5’- CTGAAGCTCACAGCCAGGA-3’
gsdf	Forward	5’- CTCGGATCAAATAGCGCAGT-3’
	Reverse	5’- TTCATCCATGAAGACGATGG-3’
3bhsd	Forward	5’- GTGCTGGAGTCGGTGGAGTA-3’
	Reverse	5’- GACATGACGTTCTCGTGGAG-3’
cyp17	Forward	5’- TGAGAAGGAGTGGACCAACC-3’
	Reverse	5’- GCTCCGAACGGCAGGTAG-3’
star	Forward	5’- GCTGCCTGCAACTTTTAAGC-3’
	Reverse	5’- TCATGATGAATGGCCACAAT-3’

rps13	Forward	5'- GTGTTCCCACTTGGCTCAAGC-3'
	Reverse	5'- CACCAATTTGAGAGGGAGTGAGAC-3'
fshb	Forward	5'- TGGAGATCTACAGGCGTCGGTAC-3'
	Reverse	5'- AGCTCTCCACAGGGATGCTG-3'

The level of expression was normalized by that of WT at the same age.

ELISA

For comparing male 11-ketotestosterone (11-KT) levels in the blood between morning and evening, we collected the blood from sexually-mature WT and *npff*$^{-/-}$ males in the morning and the evening. In addition, the blood of sexually-mature WT females was also collected in the evening. The fish were deeply anesthetized with 0.1% eugenol, and blood was collected using a borosilicate glass capillary as described in previous studies (4, 5). 0.5~ 1 μL blood was dispensed and diluted 20 times by PBS. 11-KT were extracted from diluted blood samples by using dichloromethane (Wako), and then measured by ELISA according to the manufacturer's instructions (11-ketotestosterone ELISA kit, Cat# 582751, RRID:AB_2827728, Cayman Chemical, Ann Arbor, MI). The absorbance in each well was measured at 420 nm using Multiskan FC absorption microplate reader (Thermo Fisher Scientific, Waltham, MA). We calculated the concentration of the samples by using a four-parameter non-linear regression curve fit using a computer spreadsheet provided by Cayman Chemical (https://www.caymanchem.com/analysisTools/elisa).

Retrograde neuronal tracing

The whole brain was dissected out from WT male with attaching the pituitary, and neuronal tracer Neurobiotin Plus (Vector laboratories, Burlingame, CA, USA) was injected into the pituitary by picking it using a borosilicate glass pipette of which tip was covered with Neurobiotin Plus powder. After incubation in artificial cerebrospinal fluid for 30 miunutes, the brain was fixed in 4% PFA in PBS at 4 °C for 2 hours, substituted with 30 % (w/v) sucrose (FUJIFILM Wako Pure Chemical Corporation, Osaka, Japan) in 1.0 × PBS at 4 °C for 5 hours, and embedded with 5 % ultra-low melting agarose (Sigma-Aldrich)/ 20 % sucrose (w/v) in 1.0 × PBS, and prepared cryosections by frontal cutting serially at 25 μm intervals by using a cryostat (CM 3050S; Leica Microsystems, Wetzlar, Germany).

For detecting *gpr147*, *gpr74-1*, and *gpr74-2*, *in situ* hybridization (ISH) was performed in the same methods for single ISH as described above. The sections were incubated with a horseradish peroxidase-conjugated anti-DIG antibody (Roche) diluted 1:500 with TBS-T buffer for 2 hours, washed with TBS-T, then applied Tyramide Signal Amplification / Cyanine 3 (TSA/Cy3) detection kit (Perkin Elmer, Waltham, MA) for ten minutes at room temperature.

Neurobiotin was visualized by using Alexa Fluor 488-conjugated streptavidin (Thermo Fisher Scientific) with VECTASTAIN ABC Elite Kit (Vector Laboratories) for signal amplification.

The slides were observed and photographed the region containing POm under a LSM-710 confocal laser-scanning microscope (Carl Zeiss, Oberkochen, Germany), with visualization mRNA/Neurobiotin signals by 561 nm/488 nm laser, respectively.

Statistical analysis

For comparing between WT and each knockout, all parameters were analyzed by Mann-Whitney *U* test. In case of the test for three groups (Figure. 1K), non-parametric Kruskal-Wallis test followed by Steel-Dwass multiple comparison test was conducted. Statistical

analyses and graph drawing were performed by using Kyplot 6.0 software (Kyence, Tokyo, Japan). Error bars in all bar-graphs indicate standard error of means (SEM), while the upper, middle, and lower bars in the whisker plots show the third quartile, median, and the first quartile, respectively. A *P*-value less than 0.05 was considered statistically significant.

1. C. Umatani *et al.*, Co-existing Neuropeptide FF and Gonadotropin-Releasing Hormone 3 Coordinately Modulate Male Sexual Behavior. *Endocrinology* **163** (2022).
2. A. Takahashi, S. Kanda, T. Abe, Y. Oka, Evolution of the Hypothalamic-Pituitary-Gonadal Axis Regulation in Vertebrates Revealed by Knockout Medaka. *Endocrinology* **157**, 3994-4002 (2016).
3. B. Zempo, S. Kanda, K. Okubo, Y. Akazome, Y. Oka, Anatomical distribution of sex steroid hormone receptors in the brain of female medaka. *The Journal of comparative neurology* **521**, 1760-1780 (2013).
4. M. R. Royan *et al.*, Gonadectomy and Blood Sampling Procedures in the Small Size Teleost Model Japanese Medaka (Oryzias latipes). *J Vis Exp* 10.3791/62006 (2020).
5. K. Ikegami *et al.*, Estrogen upregulates the firing activity of hypothalamic gonadotropin-releasing hormone (GnRH1) neurons in the evening in female medaka. *J Neuroendocrinol* 10.1111/jne.13101, e13101 (2022).
